# Protocol of the impact of alternative social assistance disbursement on drug-related harm (TASA) study: a randomized controlled trial to evaluate changes to payment timing and frequency among people who use illicit drugs

**DOI:** 10.1186/s12889-016-3304-6

**Published:** 2016-07-29

**Authors:** Lindsey Richardson, Allison Laing, M-J Milloy, Russ Maynard, Bohdan Nosyk, Brandon Marshall, Eric Grafstein, Patricia Daly, Evan Wood, Julio Montaner, Thomas Kerr

**Affiliations:** 1 British Columbia Centre for Excellence in HIV/AIDS, St. Paul’s Hospital, 608-1081 Burrard Street, Vancouver, V6Z 1Y6 BC Canada; 2 Department of Sociology, University of British Columbia, 6303 NW Marine Drive, Vancouver, V6T 1Z1 BC Canada; 3 Faculty of Medicine, Division of AIDS, University of British Columbia, St. Paul’s Hospital, 608-1081 Burrard Street, Vancouver, V6Z 1Y6 BC Canada; 4 PHS Community Services Society, 20 Hastings Street W, Vancouver, V6B 1G6 BC Canada; 5 Faculty of Health Sciences, Simon Fraser University, 8888 University Drive, Burnaby, V5A1S6 BC Canada; 6 Department of Epidemiology, School of Public Health, Brown University, 121 South Main Street, Providence, 02912 RI USA; 7 Department of Emergency Medicine, Faculty of Medicine, University of British Columbia, 910 West 10th Ave, Vancouver, V5Z 1 M9 BC Canada; 8 Vancouver Coastal Health, 601 West Broadway, Vancouver, V5Z 4C2 BC Canada; 9 School of Population and Public Health, University of British Columbia, 2206 East Mall, Vancouver, V6T 1Z3 BC Canada

**Keywords:** Social assistance, Drug use, Drug-related harm, Structural intervention

## Abstract

**Background:**

Government social assistance payments seek to alleviate poverty and address survival needs, but their monthly disbursement may cue increases in illicit drug use. This cue may be magnified when assistance is disbursed simultaneously across the population. Synchronized payments have been linked to escalations in drug use and unintended but severe drug-related harms, including overdose, as well as spikes in demand for health, social, financial and police services.

**Methods/design:**

The TASA study examines whether changing payment timing and frequency can mitigate drug-related harm associated with synchronized social assistance disbursement. The study is a parallel arm multi-group randomized controlled trial in which 273 participants are randomly allocated for six assistance cycles to a control or one of two intervention arms on a 1:1:1 basis. Intervention arm participants receive their payments: (1) monthly; or (2) semi-monthly, in each case on days that are not during the week when cheques are normally issued. The study partners with a community-based credit union that has developed a system to vary social assistance payment timing. The primary outcome is a 40 % increase in drug use during the 3 days beginning with cheque issue day compared to other days of the month. Bi-weekly follow-up interviews collect participant information on this and secondary outcomes of interest, including drug-related harm (e.g. non-fatal overdose), exposure to violence and health service utilization. Self-reported data will be supplemented with participant information from health, financial, police and government administrative databases. A longitudinal, nested, qualitative parallel process evaluation explores participant experiences, and a cost-effectiveness evaluation of different disbursement scenarios will be undertaken. Outcomes will be compared between control and intervention arms to identify the impacts of alternative disbursement schedules on drug-related harm resulting from synchronized income assistance.

**Discussion:**

This structural RCT benefits from strong community partnerships, highly detailed outcome measurement, robust methods of randomization and data triangulation with third party administrative databases. The study will provide evidence regarding the potential importance of social assistance program design as a lever to support population health outcomes and service provision for populations with a high prevalence of substance use.

**Trial registration:**

NCT02457949 Registered 13 May 2015.

## Background

Social assistance, including disability support, income assistance or other state-provided cash transfer benefits, is commonly distributed on a monthly basis [1, 2], and can provide important protection against the harms associated with extreme poverty [2]. Recipients of social assistance commonly increase general consumption following benefit receipt [3]. For people who use illicit drugs (PWUD), receipt of such benefits may also serve as a cue for intensified drug use [4] that is magnified when receipt is synchronized across the population [5–7]. Previous observational research has linked monthly social assistance payments to unintentional, yet cyclical and severe increases in drug-related harms [7–9], as well as increased demand for health, social, financial and police services [6–8, 10–13]. While there have been repeated calls for interventions to mitigate drug-related harms associated with synchronized social assistance [7, 8, 12], research to date has been limited to observational studies and a single natural experiment. We have therefore designed and undertaken a controlled, experimental study that examines whether changing the timing and frequency of social assistance cash transfers can reduce drug-related harm linked to the synchronization of such payments.

Socioeconomically marginalized PWUD commonly rely on social assistance as a critical source of income [14, 15]. However, the temporal synchronization of such payments has significant consequences for PWUD, their social contacts and service providers. Specifically, previous research has linked payment timing to high intensity drug use [6, 10, 16], higher risk drug use [5], increased sobering or detoxification unit admissions [7, 17], drug-related emergency department use and hospitalization [11, 13, 16, 18, 19], fatal and non-fatal overdose [5, 7, 12, 20, 21] hospital discharges against medical advice [9, 22], public disorder [7, 11], addiction and HIV treatment interruption [9, 22, 23], mental health apprehensions [24] and barriers to health service access [6]. Additionally, such increases impact demands on health, social, financial and police service provision. For example, providers have noted significant increases in patient volumes for harm reduction services such as supervised injecting sites [6], emergency department and psychiatric emergency services, police service calls and increases in cash outlays from financial service providers located in inner-city neighbourhoods (M. Corral, J. Chu, J. Fahey, personal correspondence). Challenges related to service provision are taxing for providers, interfere with their ability to provide quality and timely services and may result in individuals leaving service sites before accessing critical supports [6].

Initial documentation of drug use coinciding with cheque issue pointed to a “cheque effect”, where drug use was attributed to the provision of disability assistance [10]. A recent review examined whether disability payments are the cause of increased illicit drug use overall or whether they simply alter the timing of drug use [8]. Findings found no difference in overall rates of drug use between those receiving or not receiving disability benefits, but that payments and spikes in drug use intensity were linked. Delayed payments have produced corresponding delays in rates of drug-related hospitalization [8]. Studies provide a strong signal, first, that payments do not alter whether people use drugs, and second, that payment timing may be an important lever through which to influence payment-related increases in drug use and consequent harm. In addition, a strong rationale for leveraging pay frequency in order to smooth consumption patterns exists [25], although this has not been explored among PWUD. Smaller, more regular payments may further decrease or potentially disperse drug use and related harm. Despite indications that changing the timing and frequency of payments could significantly benefit PWUD, their communities and service providers, there is no evidence from controlled experiments identifying the impacts of such a strategy. This is a critical gap given the health, social and economic costs of drug-related harm associated with cheque issue and potentially important implications for social assistance policy.

Drug-related concerns coinciding with cheque issue may be particularly acute in inner-city neighbourhoods with higher prevalence of illicit drug use and concentrations of social assistance recipients [26]. The Downtown Eastside (DTES) neighbourhood in Vancouver, Canada is one such area, commonly characterized by: high rates of poverty; an open drug market; high prevalence of illicit drug use, mental health disorders, and HIV infection; and a large proportion of residents receiving government assistance [27]. Cheque issue days, generally on the last Wednesday of each month, are referred to locally as “Cheque Day” or “Welfare Wednesday”. The widespread and severe harms produced by synchronized social assistance are widely acknowledged among community members and service providers and have been documented by over 20 years of ongoing observational research [7, 24, 28]. The identification of public health- and community safety-promoting approaches to the disbursement of social assistance is therefore an urgent public health and community safety priority in Vancouver as elsewhere, with significant implications for policy and service provision.

We have therefore designed and initiated a prospective randomized controlled trial (RCT), the impact of Alternative Social Assistance disbursement on drug-related harm (TASA) study, to examine whether changing the timing and frequency of social assistance cash transfers can reduce drug-related harm linked to the synchronization of such payments. The TASA study is a demonstration project of the British Columbia node of the Canadian Research Initiative in Substance Misuse (CRISM). The study changes the temporal conditions of social assistance receipt to determine which disbursement arrangement most effectively mitigates escalations in drug-related health, social and economic harm associated with social assistance cheque issue days. Contrasted with behavioural interventions that seek to directly alter individual behaviour, the study is a structural intervention that alters the context (i.e., socio-economic environment) in which health risks are produced [29]. TASA focuses on both the timing and frequency of income assistance payments by comparing two distinct interventions. The first involves the disbursement of income assistance payments once a month, as is currently the case. However, payments are made on a day that does not fall during cheque issue week, and participants are randomized to different days, rather than all intervention participants receiving payment on the same non-government cheque issue day. This allows us to determine whether staggering payments separates individual cues for drug use following individual payment from the social cues for increased drug use that accompany synchronized payments from all participants being paid on the same day. The second intervention involves twice-monthly payments, similarly on days that do not fall during cheque issue week and not on the same day for all participants. In addition to differentiating between individual and social cues for drug use, this strategy tests whether splitting income assistance payments can reduce drug use by smoothing consumption patterns. Both interventions are administered through Pigeon Park Savings, a branch of Vancouver City Savings Credit Union in the DTES specifically designed to support residents who face barriers to accessing services at other financial institutions.

There are potentially important differences between the two interventions that may affect their relative efficacy. For example, staggering social assistance payments could displace consumption for individuals away from government cheque issue, but may not reduce overall consumption. At scale, this may improve experiences of service providers who would not have to manage cyclical escalations in service demand, but may provide little benefit to individual PWUD other than improved service access. Staggering and splitting income assistance payments may support smoothing consumption patterns, but may potentially result in multiple periods of escalated drug use signaled by more than one monthly payment. The testing of two different payment schedules enables us to examine which approach, if either, more effectively decreases spikes in drug use and drug-related harm coinciding with synchronized social assistance disbursement.

### Objectives

The main objective of TASA Cheque issue study is to assess the effectiveness of structural changes in the timing and frequency of social assistance payments in reducing drug use and drug-related harm in the days surrounding social assistance cheque issue in a population of socio-economically marginalized people who use illicit drugs. The interventions will be compared with each other and with a no-intervention control group.

## Methods/design

### Study design

**Fig. 1:**
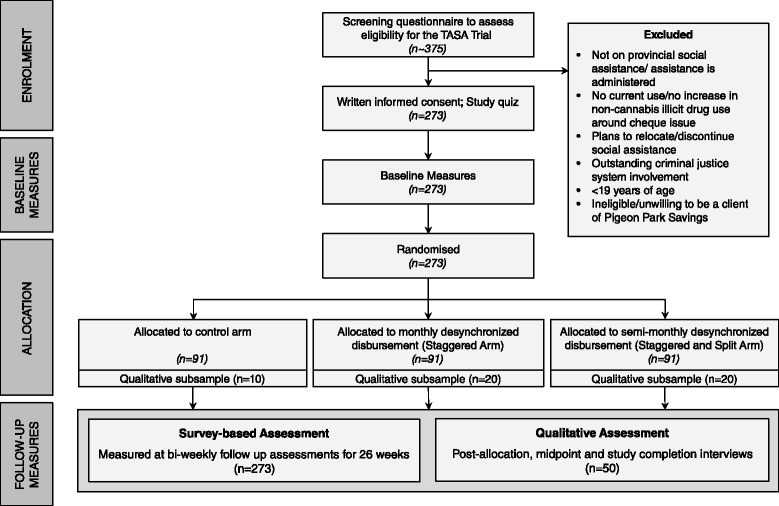
Flow of participants through the *TASA* trial

The TASA Cheque Issue study is an exploratory, parallel-group, unblinded, randomized controlled trial involving the allocation of 273 participants to either no intervention (control group), where participants continue to receive their provincial social assistance on government cheque issue day, or to one of two intervention groups: (1) monthly social assistance payment on a day that does not fall during cheque issue week (staggered group), or (2) semi-monthly on days that do not fall during cheque issue week (split and staggered group). Participants will be under active observation for six income assistance cycles, or approximately six months. The primary outcome of interest is increased frequency, street value, or number substances other than cannabis used in the 3 days starting with government cheque issue day as assessed by the standardized Timeline Followback instrument [30, 31]. The pre-specified secondary outcomes are: overall monthly drug use; increased drug use on individual cheque issue days; drug-related risk, including non-fatal overdose; barriers to service access; exposure to violence; interactions with police; emergency department (ED), emergency department mental health (EDMH) and substance use hospitalization (SUH) admissions; leaving hospital against medical advice (AMA); health care interruption or discontinuation; and changes in spending patterns as represented by the amount of time with little or no money present in the participant’s bank account. The TASA study incorporates a longitudinal nested qualitative parallel process evaluation [32] and a cost-effectiveness analysis comparing the costs of different social assistance disbursement arrangements in terms of police, judicial, corrections, crime victimization, productivity, and health care costs. The design of the trial and flow of participants are shown in Fig. 1 (Protocol version 6.6, 14 December 2015).

### Eligibility and recruitment

Individuals are eligible for the study if they are 19 years of age or older, report active and regular use of drugs other than cannabis, currently receive provincial social assistance payments on a monthly basis, report intensified drug use at the time of cheque issue days in the six months prior to recruitment, are eligible and willing to be a client of Pigeon Park Savings, and are not currently administered (where a third party manages their social assistance, often disbursing funds in smaller amounts). Cannabis is excluded from the TASA outcomes of interest and study eligibility criteria due to its lower risk profile [33] and to be consistent with standard research practice in the study context [34, 35]. Individuals are ineligible if they have imminent plans to relocate outside the greater Vancouver area or  discontinue their social assistance receipt, have outstanding criminal justice system involvement that could result in incarceration, or have been barred from membership at Pigeon Park Savings. Recruitment follows a multi-pronged approach. Three ongoing prospective cohort studies of people who use illicit drugs in Vancouver, Canada [36] have recently added the following question to their research instruments: “Did any of the following things ever happen to you in the days around cheque day?” The question is followed by a set of response options representing different types of drug-related harm, and is used to identify prospective participants for referral to the TASA study. Additional recruitment efforts utilize community-based methods, including advertisements at PWUD advocacy organizations, street-based outreach and word of mouth through the study team’s established contacts. Local health, social and financial and service providers have additionally agreed to refer clients who meet the eligibility criteria and to post advertisements for the study in their offices. The study also recruits participants through the network of addiction physicians practicing in the local health authority. Recruitment takes place on a continuous rolling basis over an 18-month period, with the final participant completing the intervention 2 years after the start of recruitment.

### Screening, consent and baseline assessment

Individuals expressing interest in the study are administered a brief screening questionnaire by study staff to determine whether they fulfill eligibility criteria. This includes confirmation that participants are willing and able to adhere to study procedures. Participants are required to provide proof of non-administered provincial social assistance receipt in the form of an official statement of benefits, recent bank statement, or cheque stub from a recent social assistance cash transfer. Additionally, government-issued identification is needed as verification of identity and to open a bank account at Pigeon Park Savings. Study procedures and requirements are then explained to potential participants to confirm participant interest and suitability.

During the consent process, research staff explain study procedures, potential risks and benefits of participation, planned data linkages to third party administrative data sets and other consent information outlined in the informed consent form. As is common for RCTs, after signing the consent form participants undergo a short oral “consent quiz” to ensure full comprehension of key points such as voluntary consent, randomization, confidentiality, study design and procedures, and withdrawal processes. Individuals receive additional information from study staff on any areas of misunderstanding identified by the quiz.

Following informed consent and prior to randomization, participants complete an interviewer-administered baseline assessment. This assessment includes the collection of demographic information and completion of questionnaire items to assess drug use, income generation and material security, drug-related activities and exposures, criminal activity, police contact, exposure to violence, drug debt, addiction treatment enrolment, health and social service use, cheque day activities and health related quality of life (see Measures section and Table 1). Baseline measures are collected for past 6-month as well as past 2-week time frames to ensure sufficient background information as well as data consistent with the 2-week time frame of follow-up observations.

**Table 1:** Measures used in the TASA Trial

			Follow-up (FU) period			Post-follow-up	
Measure(s)	Screen	Baseline	FU 1-13	Intervention withdrawal	Study exit	60-day visit	Data linkage
Primary Outcome Measure							
Daily Drug Use (TLFB)		✓	✓			✓	
Demographic Measures							
Date of Birth	✓	✓					
Gender		✓					
Ethnicity/race		✓					
Relationship status		✓					
Educational attainment		✓					
Residency / housing status	✓	✓	✓			✓	
Drug Related Activity/Exposures							
Drug use (past six months)		✓					
Expenditure on drugs		✓	✓			✓	
Binge drug use		✓	✓			✓	
Distributive and acquisitive syringe sharing		✓	✓			✓	
Crack pipe and drug equipment sharing		✓	✓			✓	
Assistance injecting		✓	✓			✓	
Public drug use		✓	✓			✓	
Non-fatal overdose		✓	✓			✓	
Addiction treatment and harm reduction							
Addiction treatment (type, timing)		✓	✓			✓	
Treatment interruptions/missed visits		✓	✓			✓	
Supervised injection facility use		✓	✓			✓	
Police contact and illegal activity							
Police contact (frequency, type)		✓	✓			✓	
Criminal activity		✓	✓			✓	
Exposure to Violence							
Exposure to violence (frequency, type)		✓	✓			✓	
Type of perpetrator		✓	✓			✓	
Police/medical involvement		✓	✓			✓	
Timing		✓	✓			✓	
Health and Social Service Use							
Service accessed		✓	✓			✓	
Barriers to service access (type, timing)		✓	✓			✓	
Missed appointments		✓	✓			✓	
Leaving hospital against medical advice		✓	✓			✓	
Income and Financial Information							
Social assistance income	✓	✓					
Additional Income Sources		✓	✓			✓	
Material security		✓	✓			✓	
Daily income generation activity		✓	✓			✓	
Banking practices		✓					
Drug Debt		✓	✓			✓	
Government cheque day activities		✓	✓			✓	
Individual cheque day activities			✓			✓	
Health-related quality of life							
Euro-QoL (EQ-5D)		✓	✓			✓	
Study-related measures							
Motivation to participate		✓					
Treatment preferences		✓			✓		
Client satisfaction questionnaire (CSQ-4)		✓			✓		
Reasons for intervention withdrawal				✓			
Duration of intervention				✓			
Participant experiences					✓		
External Data Sources							
Hospital, ED, EDMH, SUH records							
Community and primary service records							✓
Emergency health services records							✓
Supervised injection facility records							✓
Prescribed medications							✓
Banking records (VanCity)							✓
Ministry of Social Development & Social							✓
Innovation assistance receipt records							✓
Police contact records							✓

*Abbreviations*: *FU* follow-up, *TLFB* timeline follow back, *ED* Emergency Department, *EDMH* Emergency Department Mental Health, *SUH* substance use hospitalization

### Randomization, allocation concealment and blinding

Once participants complete the consent process and baseline procedures, they are randomized to one of the control, staggered, or staggered and split study arms by the study coordinator using a 1:1:1 allocation ratio. The study employs a stratified block randomization procedure [37] where randomly sized blocks comprised of equal numbers of recipients of the three main types of provincial social assistance (standard employable income assistance support, persons facing persistent multiple barriers, persons with disability) are allocated to each of the three study arms. Block sizes are random multiples of three to ensure the proportional allocation of recipients of each category of social assistance to each study arm. The randomization algorithm was developed by the study statistician in SAS software v9.4 (SAS, Cary, North Carolina, USA) and was embedded in the participant tracking system to allow for easy, on-site randomization following the completion of all study enrollment and baseline procedures and allocation concealment until randomization.

Notably, it is not possible to conceal study arm allocation from participants, given the potentially significant changes to their social assistance arrangements from the study intervention. It is also not possible to have an assessor-blinded trial, as study assessors track participant activities in relation to government as well as individual cheque issue days, thereby revealing study arm allocation information through data collection activities (see Measures section).

### Interventions

#### Control group

Participants randomized to this study arm receive no intervention. They continue to receive monthly social assistance payments according to the British Columbia Ministry of Social Development and Social Innovation disbursement schedule.

#### Staggered social assistance disbursement (staggered

Participants in the ‘staggered’ intervention study arm have access to their social assistance payments on a monthly basis on a day that does not fall during government cheque issue week and that is not the same as all other participants in the staggered arm. Due to the variation in government cheque issue day timing, which ranges from the 16^th^ to the 29^th^ of the month, individual cheque issue is allocated relative to government cheque issue day (e.g., Tuesday the week after cheque issue). Participants undergo a transition period during which they receive their social assistance up to 7 days later each month until they reach their study cheque issue date. This transition period ensures that participants do not have a time period between payments any longer than the longest period between payments on the government schedule, which is 35 days. To facilitate timely transitions to the new disbursement schedule within the timeframe of the intervention, possibilities for individual cheque issue is are limited to business days during the first and second week following government cheque issue day. This allows for 10 possible payment schedules for participants in the staggered arm. The exact date of disbursement is determined at random through a second randomization procedure. Study staff provide each participant with a detailed payment schedule to assist participant recall.

#### Staggered and split and social assistance disbursement (staggered and split)

Participants in the ‘staggered and split’ intervention study arm have their social assistance payments released twice a month on days that do not fall during the week of government cheque issue. The days of income assistance receipt are spaced 2 weeks apart and determined at random. As with the staggered intervention arm, individual cheque issue days are established in relation to government cheque issue day and participants transition to their new schedule incrementally. The first individual cheque issue day falls in the week following government cheque issue, with the second occurring on the corresponding day of the week 2 weeks later. This could be, for example, Tuesday of the week following government cheque issue day and Tuesday 2 weeks after that day. This allows for five randomization possibilities for the staggered and split intervention study arm. Participants are again provided with a schedule of their individual cheque issue days for the duration of their study participation.

The change in social assistance payment schedules for both intervention arms is managed through Pigeon Park Savings, which has developed a system, in conjunction with standard social assistance direct deposit services from the British Columbia Ministry of Social Development and Social Innovation, that varies when individuals have access to their social assistance payments. Social assistance payments are directly deposited into the participant’s account, and the system “locks” their payment so that these funds cannot be withdrawn. Money is “unlocked” according to the participant’s disbursement schedule that determines both the timing and frequency at which payments are released. Intervention arm participants can develop personalized arrangements that ensure access to essential funds at specific times to prevent housing instability or payment default. A study liaison at Pigeon Park Savings manages account creation, direct deposit requests to the Ministry and the implementation of holds and releases of funds. Accounts include unlimited withdrawals and a bank card with no-fee withdrawals at credit union automated teller machines. Pigeon Park Savings banking fees are charged at a rate of $5/month, and individuals are not required to pay this fee while they are enrolled in the study.

### Follow-up assessments

Once individuals enrol (control arm) or receive their first payment on their individual schedule (staggered and staggered and split) arms, follow-up visits occur every 2 weeks for the 26-week period of active study participation, for a total of one baseline and 13 regular follow-up interviews. Frequent follow-up visits mitigate recall-related threats to data reliability for key measures [38]. During study follow-up visits, a trained interviewer administers the follow-up questionnaire, which includes past 2 week assessments of all study measures. Participant safety, including the monitoring of adverse events or serious adverse events related and unrelated to study participation is also assessed at every follow-up. Follow-up visits take 20–30 min. Due to the intent-to-treat nature of the study, participants can withdraw from the intervention but are encouraged to continue to complete regular follow-up visits.

### Qualitative parallel process evaluation

The TASA study also includes a qualitative parallel process evaluation concurrent to the quantitative survey-based evaluation of the study interventions. This evaluation is comprised of a longitudinal, nested study involving a sub-population of TASA study participants who complete three qualitative interviews each, the first at baseline, the second at their study mid-point and the third at study completion or withdrawal. Following randomization to the study interventions, approximately 45–50 participants are selected to be participants in a qualitative parallel process evaluation. Quota sampling ensures heterogeneity across categories of social assistance, drug use practices and study arms, with a weighted distribution focusing on the experiences of individuals allocated to the intervention arms (8–10 participants recruited from control versus 15–20 from each intervention arm). Following a separate informed consent process specific to qualitative participants, audio-recorded interviews are conducted by trained qualitative interviewers in private settings at the study site. Data collection follows principles of data saturation in which the study team continues to recruit participants until no new themes emerge in relation to key topics from interviews among individuals in each of the study arms [39, 40]. Interviews employ topic guides informed by previous studies conducted by the investigative team [5, 6, 41] and a comprehensive review of the relevant literature. Baseline interviews explore prior experiences of social assistance receipt, income and material hardship, substance use patterns, and financial management. Midpoint interviews focus on transitions to new payment schedules as well as changes in material circumstances, financial management, drug use patterns and expenditure on drugs. Exit interviews explore study experiences of social assistance receipt and broader changes to life circumstances (e.g. income, housing), drug use and drug-related activity, and reasons for study or intervention withdrawal, where applicable. Initial interviews inform subsequent interviews among the same participants, building on previous responses and benefits or challenges previously identified by participants.

### Study completion and post-evaluation questionnaires

At the final regular follow-up study visit, participants are administered a study completion questionnaire that assesses, in addition to regular follow-up measures, their participation experiences and opinions about the intervention. Sixty days post study completion, a follow-up interview is administered to assess the safety of participants and any significant changes following the completion of the study protocol. Participants in all study arms are able to access the study intervention of their choice following the completion of their participation should they wish to do so. Screening, baseline, follow-up, qualitative and post-evaluation study visits take place at a purpose-built field research office located in the DTES and operated by the British Columbia Centre for Excellence in HIV/AIDS. If needed, follow-up visits can take place at the participant’s residence, over the phone, or other space considered safe by the participant.

### Participant honoraria

Consistent with standard practice in research involving PWUD [34, 42], participants are compensated for their time and interview-related expenses with honoraria in the following Canadian dollar (CAD) amounts: $30 for baseline/randomization visit; $10 per follow-up interview, with follow-up interview incentive bonuses following the first post-baseline follow-up ($10), the completion of five follow-up interviews ($15), the completion of nine follow-up interviews ($20) and the completion of the final follow-up interview ($25). The post study follow-up interview honorarium is $15. The honorarium for each qualitative interview is $30 per interview. Participants will therefore receive a maximum of $245 for participation in the quantitative portion of the study and $335 for participation in both the qualitative and quantitative study components.

### Measures

Table 1 outlines the measures taken at screening, baseline, follow-up, withdrawal and study exit interviews and through post-study data linkages. For all primary and secondary outcomes of interest described below, we collect data on indicators on government cheque issue days by tracking activity and exposures on a daily basis or through specific questionnaire items and response options. Where government cheque issue and individual cheque issue timing are not the same (i.e., for intervention participants), we additionally ask specific questions about individual cheque issue day activities and exposures. By collecting information about the occurrence of key outcomes of interest on non-cheque issue days as well as those coinciding with government and individual payments, we are better able to distinguish between individual cues for increased drug use coinciding with individual payments and social cues prompted by synchronized payment across the population on government cheque issue day.

#### Socio-demographic information and housing status

Socio-demographic information, collected at baseline, includes standardized measures of age, gender, ethnicity, relationship status and highest level of educational attainment. Self-reported measures of residency and housing status include neighbourhood of residence, type of residence (e.g. house, apartment, single room occupancy hotel [43], no fixed address), and housing stability, measured by the number of residences occupied in the last six months (baseline) or past 2 weeks (baseline and follow-up).

#### Drug use (timeline follow back; primary outcome measure)

The TASA study’s primary outcome is increased intensity of drug use on government cheque issue day (i.e. a binary outcome). Participant drug use is considered to have intensified if, in the 3 days starting with government cheque issue day, a participant increases by 40 %: (1) the daily frequency of non-cannabis drug use; (2) the street value of drugs consumed; or (3) the number of non-cannabis substances used, including alcohol and illicit prescription opioids, all compared to the average frequency, value of drugs used or number of substances used on all other days of the calendar month. For example, if an individual uses crack cocaine once daily on non-cheque issue days and does not use methamphetamine, but increases uses crack cocaine 3 times per day and methamphetamine once per day on the 3 days beginning with cheque issue day, they will have intensified their use according to the first and second criteria. These cut points were selected based on prior empirical studies [8] and our intention to capture changes among high-intensity users that could be overlooked if more blunt measures common to RCTs among PWUD, such as daily drug use [44], are used. Staff gather data measuring the primary outcome using the Timeline Follow Back (TLFB) instrument, a reliable, validated, calendric instrument that collects daily information on drug use patterns, including drugs used and frequency of use [30, 31]. We additionally collect participant estimates of the street value of drugs used as a proxy for quantity or dosage of drug consumed, which may not be captured by drug use frequency measures [45, 46]. We also use daily TLFB drug use data to assess our key secondary outcome of intensified drug use on individual cheque issue days, identified using the same frequency, street value or number of drugs criteria as intensified drug use on government cheque issue day.

#### Drug-related activities and exposures

A range of activities and exposures has been associated with increased risk of drug-related morbidity and mortality as well as social and community harms [47–51]. To support analyses of secondary outcomes of interest, past six month (baseline) and past 2 week (baseline and follow-up) self-reported measures for drug use-related activities and exposures include: changes in drug use patterns (e.g., binge or greater than average drug use); distributive and acquisitive syringe, crack pipe or drug equipment sharing; receiving an assisted injection; public injection and non-injection drug use; and non-fatal overdose. Questionnaire items have previously been verified for use in the current study context through longstanding cohort studies involving PWUD [52].

#### Addiction treatment enrolment and harm reduction service use

Past six month (baseline) and past 2 week (baseline and follow-up) data on addiction treatment enrolment are collected for secondary analyses about addiction treatment interruption. Questions include the type of addiction treatment, interruptions to ongoing opioid assisted or other treatments, missed visits, the reasons for addiction treatment interruption (if any), and the impacts of treatment interruptions or missed visits (e.g., relapse, withdrawal). We additionally ask participants about past six month (baseline) and past 2 weeks (baseline and follow-up) use of Insite, a local supervised injection facility (SIF), given the association between exposure to the SIF and significant improvements in in morbidity, mortality, uptake of addiction treatment uptake and measures of public order [53].

#### Police contact and illegal activity

While drug market enforcement has been shown to have adverse public health and social impacts [54], studies have also identified police as critical supports for public health and community safety initiatives [55]. We collect data on all police interactions, and the measures used in the current study facilitate distinctions between different types of police contact (e.g., service referral, arrest), as well as participant perceptions of the reason for contact. In support of economic analyses, we also collect data about engagement in illegal activity to assess any changes in the costs of criminality and victimization as a result of the intervention, with past six month (baseline) and past 2 week (baseline and follow-up) recall periods.

#### Exposure to violence

Consistent with previous analyses documenting the relationship between socio-economic marginalization and exposure to violence [56], the TASA study also seeks to document whether varying social assistance payments impacts participants’ exposure to violence. The study instrument includes measures to identify the frequency, timing and type of violence victims were exposed to, the type of perpetrator (e.g. stranger, intimate partner) and whether the participant sought police or medical attention. The study solicits analogous information regarding the perpetration of violence.

#### Health service utilization

Several key secondary outcomes of the TASA study are linked to whether the study intervention can reduce or smooth health service utilization. Previously identified health service impacts of synchronized social assistance include ED, EDMH or SUH admissions [7, 11–13, 19, 57]; discharges against medical advice directly prior to cheque issue [7, 9, 12]; health service access barriers linked to elevated demand [6]; and interruptions to ongoing medical care [9, 22]. In support of these assessments the study instrument collects past six months (baseline) and past 2 weeks (baseline and follow-up) information on which health services were accessed, whether participants attempted but were unable to access a service, the timing and reason for encountering service access barriers and whether participants missed medical appointments around cheque issue. We additionally collect information on whether, if hospitalized, participants left hospital against medical advice, as well as the reasons for and timing of their departure.

#### Social service utilization

Social assistance recipients’ use of social services, such as meal programs, outreach services or drop-in centres, may similarly undergo cyclical changes or face barriers to accessing services at times when these are oversubscribed. Similar to health care utilization indicators, the study instrument includes questions asking participants about social service access, barriers to accessing services and the timing of service access barriers in relation to cheque issue in the past six months (baseline) and past 2 weeks (baseline and follow-up).

#### Income and material security

Consideration of income generation is critical given that many social assistance recipients supplement their income with illegal or prohibited income generation (e.g., sex work, drug dealing) [14, 15], and that such activities are associated with health harms [14, 15, 56]. Therefore, changing assistance patterns may change patterns of other income generation. To assess the impact of the intervention on financial well being, we measure income generation activity, income amounts and material security in the past six months (baseline), and past 2 weeks (baseline and follow-up). Data referring to the past six months is collected using an instrument verified for use in the current study context [15, 58]. Data referring to the 2 weeks prior to interview is collected by expanding the use of the Timeline Followback instrument to track daily income generation information, including sources and estimated daily earnings. Material security in the past six months (baseline) and past 2 weeks (baseline and follow-up) is assessed using a validated scale [59] that has been adapted to the current study context. Further measures include involvement in acquisitive criminal activity, such as theft, as well as amounts of drug debt accumulation and to whom drug debt is owed.

#### Quality of life

To support health economic evaluation, the Canadian Euroqol Group EQ-5D [60, 61] will be administered at baseline and all follow-up visits. The EQ-5D can be administered quickly and measures health related quality of life and includes participant self-reports of mobility, self-care, usual activities, pain/discomfort and anxiety/depression, as well as a health thermometer ranking of a participant’s overall state of health, and has been validated for use among PWUD [62].

#### Study-related measures

To assess considerations related to the study and study intervention that may affect study outcomes, at baseline we collect information on individual’s motivations to participate in the study (e.g., financial compensation, desire to reduce drug use around cheque issue) and their treatment arm preference. At baseline and the final follow-up visit, participants complete the client satisfaction questionnaire (CSQ), a validated instrument [63] that assesses satisfaction with services and is used here to measure participant perceptions about social assistance receipt, including the degree to which social assistance and related Ministry of Social Development and Social Innovation supports meet needs, help with problems, and are satisfactory. Additionally, for participants that withdraw from the intervention prior to completing their 26-week intervention period, questions are asked about their reasons for and circumstances surrounding withdrawal. At the final study follow-up we assess participant experiences, including participant opinions about the benefits and drawbacks of their intervention and best options for cheque issue timing and frequency.

### Participant safety

The design of the current trial does not involve any change in the amount of social assistance provided to participants, and we therefore do not anticipate any negative changes to their financial well-being. However, desynchronizing social assistance may disrupt money management strategies, potentially interfering with the payment of expenses. Importantly, most participants in the TASA study have the rent portion of their social assistance paid directly to their landlords. Participants who have rent or other expenses can make arrangements on a case-by-case basis with Pigeon Park Savings to ensure the availability of funds at the appropriate time. Additionally, for staggered and split arm participants, receiving multiple payments per month may result in more frequent periods of intensified drug use, albeit these periods will likely be shorter or less intense given that less money will be disbursed at any one time. For intervention participants, there may also be challenges transitioning onto or off of a new social assistance payment schedule at the start or end of the study period. To mitigate this concern, study staff members can provide referrals to financial management support services as appropriate. We do not anticipate the study will expose participants to unusual risks. Nevertheless, we monitor participant safety as a part of each bi-weekly participant assessment, including considerations of food and housing insecurity, exposure to violence, access to critical health and social services and other emerging issues. We follow standard reporting procedures for adverse events and serious adverse events [64], including oversight from an external, independent Data Safety and Monitoring Committee (DSMC). Participants are able to withdraw from the intervention, the study, or both at any point, and may be withdrawn from either by investigators in the event of concerns over safety.

### Sample size

We base sample size calculations for the TASA study on an a priori minimum clinically important difference (MCID) in the rate of individuals reporting intensified drug use on government cheque issue days between control and non-control participants. For the purposes of sample size calculations, comparisons were drawn between control and staggered arm participants. We anticipate this intervention will have a lesser impact on the primary outcome of interest than the staggered and split arm and should therefore provide basis for sample size calculations. Given a lack of prior comparable experimental research, the MCID was set conservatively to a 20 % difference between control and staggered intervention arm participants. This MCID was based on results from previous observational studies that report increases in the rates of drug related harm between 24.9 and 81.2 % in the days surrounding government cheque issue [5, 7, 12, 13, 17–20, 57]. We combined this MCID with an estimate of 85 % of individuals in the control group reporting intensified substance use on community cheque issue day (*Pc*). This was set lower than 100 % to account for potential reporting biases at study screening, where all participants must indicate elevated use in order to be eligible, as well as any study participation effect on the outcome of interest among control arm participants.

Sample size calculations used methods to detect differences between proportions in a repeated measures design and were calculated using Power and Sample Size (PASS v.12) software. Sixty-five participants per arm completing all follow-ups would allow the detection of a 20 % difference in the rates of intensified drug use between control and staggered intervention arm participants with 80 % power at a 5 % significance level. These calculations assumed that control and intervention arm rates of intensified drug use follow a Bernoulli distribution, the number of government cheque issue days falling within the observation period is 6 and, as in previous studies involving PWUD [65], the autocorrelation between measures of the same individual over time *(ρ)* is 0.6. Based on previous studies among PWUD, and noting the importance of accounting for missed follow-up visits and loss to follow-up [66], we additionally account for observation-level non-response of 33 % and a potential rate of loss to follow-up of 25 %, reaching a final sample size of 91 individuals per arm or 273 in total across all three arms. The possibility of type II, false negative findings, could result from insufficient power, or, in the event of intervention withdrawal, the dilution of the effect of the intervention in ITT analyses from participants who have resumed regular synchronized payments but are analyzed as part of the intervention arm. These risks will be partially mitigated by the inclusion of sensitivity analyses, as outlined in the Data Analysis section below.

### Data linkages

To verify participant self-report wherever possible, and supplement data for the assessment of cheque issue-related costs incurred by institutions that provide social, health, financial and security (i.e., policing) services to PWUD and their communities, we will obtain third party service provision and administrative records from external databases on all participants. Participant records will be requested from hospitals, health service providers, the provincial prescription medication database, Provincial Ministry of Social Development and Social Innovation, study partner credit union, and police and criminal justice databases. Records will be requested for a 30-month period beginning 1 year before the beginning of active study participation through to 1 year after following the completion of active study participation in order to assess the impact of study involvement on access to medical and social services over time. Data linkages will be established through participant names, birth dates and participants’ personal health number, a unique and persistent identifier issued for medical billing and tracking purposes to all residents of British Columbia. Data will be de-identified to protect participant confidentiality.

### Data analysis

#### Quantitative analyses

At the conclusion of the trial, the primary and secondary endpoints will be assessed via intention-to-treat analyses [67], including all patients randomized regardless of compliance with the intervention protocol. Sub-analyses will include a modified intention-to-treat analysis that will use all participants with at least one follow-up [67, 68]. First, we will compile descriptive characteristics of the sample, assessing for systematic differences in key characteristics across study arms using the Chi-Square test for binary variables and the Mann-Whitney *U* test for continuous variables. The primary outcome of interest will be derived from TLFB information as a repeated binary variable (intensified drug use on the 3 days beginning with government cheque issue day vs. not) and will be quantified as the proportion of individuals TASA participants reporting increased drug use during this period at each government cheque issue. This outcome, and other secondary outcomes similarly quantified will be examined using pooled generalized linear mixed effects models, with binomial distribution and logit link specified to produce unadjusted and adjusted odds ratios and 95 % confidence intervals. Other secondary outcomes of interest, quantified as counts of the number of occurrences over the course of the observation period (e.g., hospital ED admissions), will be examined using generalized linear mixed-effects model, with a Poisson distribution and a log link specified for count data with corrections for overdispersion, including a Vuong test for overdispersion and, where indicated, the use of a negative binomial model [69]. All analyses will include binary indicators of study arm allocation to assess for systematic differences in primary and secondary outcomes of interest across study arms. Multivariate analyses will be adjusted for: (1) binary indicators of the type of income assistance the participant receives; (2) an indicator variable for whether the individual changed their financial service provider to Pigeon Park Savings (PPS) at the start of the study to control for the effect of becoming a PPS client; (3) socio-demographic characteristics, including gender, ethnicity and age; indicators of addiction treatment enrollment and DTES residency, and (4) a categorical variable for the number of days between government cheque issue and their study visit to account for differences in recall reliability. As is common in RCT analyses [70, 71], missing assessments of intensified drug use will be treated as positive and tested for sensitivity to this assumption [72, 73]. Additional sensitivity analyses will include: (1) a modified intention-to-treat analysis using all participants with at least one follow-up assessment [74]; and (2) per-protocol analyses of participants who maintain enrollment in the intervention [75, 76].

#### Qualitative analysis

Following verbatim transcription of interview audio-recordings, we will inductively generate a coding framework capturing a priori key analytic constructs derived from the topic guide as well as emergent themes derived from interview transcripts, continuously refining and consolidating code categories. Qualitative data will be contextualized through linkages to TASA questionnaire data in terms of individual participation trajectories, adverse events and serious adverse events. Data from second and third interviews will test the boundaries of existing categories where overlap in substantive content exists, identify new conceptual categories and examine negative or inconsistent evidence. Particular focus will be on experiences of synchronized and unsynchronized cheque issue days among intervention participants to explore the role of individual and social cues for drug use. We will also compare experiences of drug use, drug-related harm and changes to financial management strategies between individuals allocated to different intervention arms. Longitudinal data collection will provide opportunities for participants to provide feedback on emerging analyses and reflect on earlier study experiences.

### Economic evaluation

The economic evaluation of the TASA study will involve a cost-utility analysis to determine the cost-effectiveness of the study interventions. We will adopt a societal perspective that includes the direct (healthcare utilization: medication, inpatient and outpatient care) and indirect (criminal activity and lost productivity) consequences of different social assistance disbursement arrangements. Incremental cost-effectiveness ratios (ICERs) will be calculated for the 26-week timeframe of individual using trial-based analysis, contrasted with results from a model-based analysis projecting up to a lifetime horizon by adapting a previously-developed model [77]. Statistical analyses will include: (1) Cost estimation; (2) Health-related quality of life (HRQoL) assessment and valuation; (3) Incremental cost-effectiveness analysis; and (4) Analysis of uncertainty. The relevant costing data includes intervention costs, health resource utilization and criminality, all collected at follow-up via standardized or verified instruments, with previously-used unit cost sources [77]. The number of quality-adjusted life-years (QALYs) [78] accumulated during the study period for each individual will be estimated using the Euroqol EQ-5D [62]. We will augment standardized measurement by quantifying QALY loss due to overdose via direct assessment at baseline.

ICERs will be calculated in the typical formulation, for both the trial- and model-based analyses [79]. Additionally, we propose a net-benefit regression approach for the trial-based analysis, using established econometric techniques [80], allowing for subgroup assessment of cost-effectiveness. Uncertainty around ICERs will be quantified using non-parametric bootstrapping and Monte Carlo simulation for trial- and model-based analyses, respectively [81]. This information can then be used to inform whether it is worthwhile to collect further information to clarify the decision rule, in the event that there is no clear choice among control or intervention strategies, or if there is a high level of uncertainty in the decision [82]. The analysis will conform to guidelines on cost-effectiveness analyses conducted alongside clinical trials [77, 83].

### Study oversight

TASA is monitored by an independent DSMC comprised of a biostatistician, epidemiologist, addictions physician and community service provider with detailed knowledge of the interests of the target study population. The DSMC can recommend study stoppage for reasons of efficacy, recruitment or participant safety, and meets semi-annually. Data concerning adverse events and serious adverse events are provided in real time, and study investigators submit quarterly reports on participant recruitment and retention. Interim analyses whose results are to be reported to the DSMC are scheduled following the recruitment and study completion of the first third and second third of study participants. Any protocol modifications will be subject to further approval form the ethical review and reported to all study investigators, trial registries, journals and, where relevant, trial participants.

### Data management and quality assurance

Each participant is assigned a unique numeric study identifier code at the beginning of their enrollment in the TASA trial to enable the de-identification of data. Confidential electronic questionnaire data and completed hard copy forms will be entered into a password-protected Oracle database. Qualitative data stored in electronic files, including interview recordings, interview transcripts as well as field-notes will be password protected. Hard copies of field notes, consent forms, and interview transcripts will be stored in locked filing cabinets in the secure field research office in space restricted exclusively to study staff and maintained by study investigators. All participant data and digital research documents are stored in encrypted files on secure servers at the British Columbia Centre for Excellence in HIV/AIDS, and only the Principal Investigator and key study personnel have access to any data containing personal identifiers to facilitate participant safety and monitoring and data linkages. Study personnel perform random checks to verify the accuracy of data input into the clinical trial database, and triangulation will be undertaken to identify and rectify any deficiencies to ensure data integrity. Trained personnel conduct the TASA study according to standard operating procedures and the principles of Good Clinical Practice.

## Discussion

The unintended health, social and economic harms resulting from synchronized monthly social assistance are well documented [5–7, 9–13, 16–24]. However, the lack of controlled experimental studies examining alternate disbursement strategies impedes the development of evidence-informed policies that could preserve the important financial security and other benefits of social assistance while decreasing their role in the production of drug-related harm. The TASA study is the first RCT that begins to address the significant gap in our understanding of potential alternatives to a considerable driver of avoidable morbidity and mortality, elevated costs to communities and challenges to service provision. By providing robust research exploring interventions to address both the individual and social effects of current disbursement policies, the study is being conducted with a view to identify strategies that are better able to promote public health and safety. This study therefore has considerable implications for policy makers given the longstanding and widespread recognition of disbursement-related harm in multiple jurisdictions [7, 8, 21, 84].

The study also represents a notable innovation in experimental and trial-based research in addictions by focusing on a structural intervention that alters the characteristics of an upstream determinant of health-individual income-to mitigate the effects of problematic drug use and related harms. RCTs among PWUD have overwhelmingly assessed behavioural drug use interventions, and have, as a result, produced mixed evidence for interventions that may be difficult to scale up or that produce only short-term benefits [85–87]. In contrast, the TASA study examines an intervention with significant policy relevance and a high degree of scalability, with the possibility for long-term, low-cost or cost-averting implementation. Additionally, the measurement of daily drug use patterns throughout the 26-week duration of each participant’s active study participation produces a robust platform for analyses. The supplementation of study data with information from third-party databases will additionally allow for data triangulation and robust service utilization and cost-benefit analyses to determine whether either of the tested interventions holds the potential to produce system-wide efficiencies and reduce service provision challenges around government cheque issue. The study additionally benefits from widespread community acknowledgement of the issues and challenges of synchronized social assistance and strong community partnerships in support of the study and the assessment of alternative approaches.

The TASA study uniquely examines an upstream determinant of health and the unintended but negative consequences of public policy on the outcomes of vulnerable and marginalized populations. In testing changes to the structural conditions of social assistance receipt for PWUD through a community-based RCT, the TASA study advances the field of experimental addictions research. The inclusion of a paired nested qualitative parallel process evaluation and economic cost-effective analyses provide a robust platform for understanding the impacts, mechanisms of action and cost implications of alternative approaches to synchronized monthly social assistance disbursement. There is significant potential, therefore, for TASA study findings to directly support evidence-informed changes to social assistance disbursement policy in British Columbia, Canada and internationally.

## Abbreviations

AMA, against medical advice, CAD, Canadian dollars, CRISM, Canadian Research Initiative in Substance Misuse, CSQ, client satisfaction questionnaire, DSMC, Data Safety and Monitoring Committee, DTES, Downtown Eastside, ED, emergency department, EDMH, emergency department metnal health, HRQoL, health-related quality of life, ICER, Incremental cost-effectiveness ratios, PPS, Pigeon Park Savings, PWUD, people who use illicit drugs, QALY, quality adjusted life yearsRCT, Randomized controlled trial, SIF, supervised injection facility, SUH, substance use hospitalization TLFB, Timeline follow back instrument
